# Phylogenetic Relationships of Avian Cestodes from Brine Shrimp and Congruence with Larval Morphology

**DOI:** 10.3390/ani14030397

**Published:** 2024-01-25

**Authors:** Stella Redón, Mauricio Quiroz, Dunja Lukić, Andy J. Green, Gonzalo Gajardo

**Affiliations:** 1 Departamento de Biología Vegetal y Ecología, Facultad de Biología, Universidad de Sevilla, Av. Reina Mercedes, 41012 Seville, Spain; 2 Departamento de Ciencias Biológicas y Biodiversidad, Universidad de Los Lagos, Av. Fuchslocher 1305, Osorno 5290000, Chile; mjquiroz@gmail.com (M.Q.); ggajardo@ulagos.cl (G.G.); 3 Department of Conservation Biology and Global Change, Estación Biológica de Doñana, EBD-CSIC, C/Américo Vespucio 26, 41092 Seville, Spain; dunjalkc@gmail.com (D.L.); ajgreen@ebd.csic.es (A.J.G.)

**Keywords:** Platyhelminthes, Hymenolepididae, ribosomal 18S, Anostraca, waterbirds, molecular phylogeny, phylogeography

## Abstract

**Simple Summary:**

Hypersaline lakes provide services like nest sites and food for migratory waterbirds. The crustacean *Artemia* (brine shrimp) is an intermediate host for many cestode parasites completing their life cycle in multiple waterbirds (flamingos, grebes, waders, and ducks) associated with the hypersaline lakes. The molecular identification of larval stages of six cestodes in *Artemia* from three countries correlated well with the morphology of advanced larval stages. This study provides the first parasite molecular data on *Artemia*-cestode-bird relationships to facilitate our understanding of food web dynamics and service provisioning in hypersaline lakes, which are threatened by human-caused perturbations, including climate change.

**Abstract:**

Determining molecular markers for parasites provides a useful tool for their identification, particularly for larval stages with few distinguishable diagnostic characters. Avian cestodes play a key role in the food webs and biodiversity of hypersaline wetlands, yet they remain understudied. Using naturally infected *Artemia*, we identified cestode larvae (cysticercoids), assessed their genetic diversity, and explored phylogenetic relationships in relation to larval morphology and waterbird final hosts. We obtained partial 18S rDNA sequences for 60 cysticercoids of the family Hymenolepidae infecting *Artemia* spp. from seven localities and three countries (Spain, the USA, and Chile). We present the first DNA sequences for six taxa: *Confluaria podicipina*, *Fimbriarioides* sp., *Flamingolepis liguloides*, *Flamingolepis* sp. 1, *Flamingolepis* sp. 2, and *Hymenolepis californicus*. Intraspecific sequence variation (0.00–0.19% diversity) was lower than intergroup genetic distance (0.7–14.75%). Phylogenetic analysis revealed three main clades: 1—*Flamingolepis*, 2—*Fimbriarioides*, 3—*Confluaria* and *Hymenolepis*, all of which separated from hymenolepidids from mammals and terrestrial birds. This clear separation among taxa is congruent with previous morphological identification, validating the 18S gene as a useful marker to discriminate at generic/species level. Working with intermediate hosts allows the expansion of knowledge of taxonomic and genetic diversity of cestodes in wildlife, as well as elucidation of their life cycles.

## 1. Introduction

Parasites have crucial roles in ecosystem functioning and services, and are important sources of information on the ecology of their hosts [1]. However, knowledge of the diversity of endoparasites of aquatic birds, such as cestodes, remains limited [2]. Studies aimed at delimiting parasitic species are mostly based on both the morphology of adult specimens and life cycle characteristics. Larval stages have a limited number of morphological features and their identification can be problematic. However, working with adult parasites implies post-mortem examination of the final hosts, which can be restricted for waterbirds, particularly protected species such as South American flamingos, which are affected by climate change and mining impacts [3,4].

Accurate identification of larval parasites is relevant to understanding the life cycle of parasites, host–parasite interactions, disease transmission and diagnosis (reviewed by [5]), and for a more comprehensive knowledge of global biodiversity [6]. Molecular tools are increasingly used in parasitological studies and can help identify the final hosts of parasites, detect them, and characterize their communities [7,8]. However, there are important biases in the research on helminth parasites related to specific taxa and geographical areas [9]. In addition, parasites in wildlife have received little attention compared to taxa with medical, zoonotic, or veterinary importance [9].

Cestodes are a group of obligate helminth endoparasites with multiple-host life cycles and trophic transmission through predator–prey interactions. Adults of most species parasitize mammals, but others have fish or birds as final hosts. The life cycle includes an invertebrate as intermediate hosts in which the parasite develops to an infective larval stage ready to reach the vertebrate host, where it matures and reproduces. Although cestodes are involved in major diseases in humans and wildlife, knowledge of their biodiversity is limited, being particularly scarce for those infecting wild aquatic birds in the Neotropics [2,10]. Flamingos are major hosts, yet the cestode fauna of flamingos from South America is currently understudied. A unique cestode (*Flamingolepis chilensis*) was described from the Andean flamingo *Phoenicoparrus andinus* [11], but there is no previous information for the Puna flamingo *Phoenicoparrus jamesi* and the Chilean flamingo *Phoenicopterus chilensis*. Early stages of parasites (i.e., eggs and larvae), obtained from fecal samples or intermediate hosts, can be used to study parasite biodiversity, host associations and biogeography, overcoming methodological limitations of working with vertebrate final hosts. Recent morphological studies of larvae in crustacean intermediate hosts (*Artemia* spp.) from Chile provided evidence of additional *Flamingolepis* species, as well as the first reports of the progynotaeniid *Gynandrotaenia stammeri* from South American flamingos [10,12].

The application of molecular techniques to helminth parasites can discover cryptic parasite species [13,14], characterize larval cestodes [15,16], and reconstruct phylogenetic relationships [17,18,19]. The genes encoding the nuclear ribosomal RNA are widely employed for diagnosis of cestodes infecting mammals [20,21,22,23]. In the last decade, large and small subunit rRNA gene (i.e., 28S and 18S) have been used to explore the phylogeny within the cestode family Hymenolepididae Ariola, 1899 (the most diverse with 923 recognized species, [2]), with emphasis on mammalian parasites [24,25,26,27,28,29].

The brine shrimp *Artemia* spp. (Crustacea: Branchiopoda: Anostraca) has a key role in the life cycle and transmission of avian cestodes in hypersaline lagoons, lakes and coastal salterns [10,30,31,32,33,34], which are valuable habitats and breeding grounds for resident and migratory birds (e.g., in South America: [3,35,36]). It serves as an intermediate host for at least 22 cestode taxa, 15 of them hymenolepidids [37], parasitizing flamingos, grebes, waders, gulls, and ducks [12]. Three putative cestode species new to science have recently been recorded in South American *Artemia* populations [10,12]. However, all studies on *Artemia* parasites in any continent are based on morphological and morphometric data obtained from microscopic examination, without any genetic information.

Here, we present the first study to molecularly identify avian cestodes recorded in brine shrimps. We sequenced 18S rDNA gene fragments of hymenolepidid cestodes infecting *Artemia*, including specimens from localities in the Nearctic, Palaearctic, and Neotropical realms. We focused on the Hymenolepididae family since it is the most speciose and prevalent in brine shrimp hosts, and includes parasites of different waterbird families. Our main objectives were firstly to compare the genetic diversity of larvae with their diversity according to morphology, to establish the extent to which a molecular phylogeny is consistent with taxonomy based on larvae morphology, and secondly, to estimate genetic variation within morphospecies recorded in different continents, and/or in different host species. We compared sequences for larvae assigned to the six morphospecies from the genera *Confluaria* (parasite of grebes), *Fimbriarioides* (parasites of ducks), *Flamingolepis* (parasite of flamingos), and *Hymenolepis* (parasites of Charadriiforms), including species yet to be fully described. We looked for cryptic species, i.e., extensive sequence divergence in cases where the same morphospecies has been described in different biogeographical realms. We discussed our results in relation to the movement ecology of bird species acting as final hosts.

## 2. Materials and Methods

### 2.1. Artemia Samples and Parasite Material

*Artemia* samples were collected between 2007 and 2018 in several hypersaline wetlands from Europe and the Americas as part of different research projects. Samples from seven localities and three countries were used in the present study, including the asexual *Artemia parthenogenetica* from two coastal salterns in Spain (Bras del Port salterns in Alicante province and San Pedro del Pinatar salterns in Murcia province), and two sexual species: *Artemia franciscana* from an inland lake in USA (Great Salt Lake in Utah) and from two lagoons in Northern Chile (Tebenquiche lagoon and Barros Negros lagoon in Salar de Atacama, Region of Antofagasta) and *Artemia persimilis* from two lagoons in the south of Chile (Amarga lagoon and Los Cisnes lagoon in Patagonia, Region of Magallanes and Chilean Antarctica).

A total of 8262 *Artemia* individuals were examined under the microscope for cysticercoids in previous works (see [10,12,33,38] for details of sampling and fixing procedures). From those, 120 infected brine shrimps (45 *A. persimilis*, 58 *A. franciscana*, and 17 *A. parthenogenetica*; see Table S1) were preserved in absolute or 70% ethanol at 4 °C for molecular analysis. Some cysticercoids of *Flamingolepis liguloides* (bigger size) were isolated from the *Artemia* tissue and fixed in absolute ethanol individually and in pools of 2–5 cysts (Table S1). This allowed us to test if parasite isolation and/or the quantity of material increased the quality of DNA and thus the 18S amplification. Most DNA extractions were performed on an *Artemia* individual infected with one cysticercoid (see Table S1). Indeed, a single *Artemia* infected with one cestode cysticercoid was enough for positive amplification (independently of the cysticercoid size), indicating that isolation of parasites from the *Artemia* tissue was not essential for a successful PCR reaction. Pools of 3 *Artemia* individuals from Los Cisnes lagoon infected with *Flamingolepis* sp. 1 (the most prevalent parasite in the south of Chile) were also used in the analyzes (N = 4), but no differences in the results were obtained compared to individual samples. Parasites isolated from individuals with multiple infections (i.e., with more than one cestode species) were used for the less abundant cestode taxa.

Cysticercoids were morphologically identified as *Confluaria podicipina, Fimbriarioides* sp., *Flamingolepis liguloides*, *Flamingolepis* sp. 1, *Flamingolepis* sp. 2, and *Hymenolepis californicus* (after [10,12,30,33,38,39]). Identifications were mainly based on the size of the cysts, type of rostellum, number, size and shape of rostellar hooks, and length of cercomer (see above references for more details). Three uninfected *Artemia* individuals from Los Cisnes lagoon collected in November 2017 were considered as negative control for the PCR reaction, allowing us to exclude non-specific *Artemia* genomic DNA amplification.

### 2.2. Genomic DNA Extraction

Genomic DNA was extracted from infected *Artemia* individuals preserved in ethanol using phenol chloroform-isoamyl (see details in Appendix A). DNA samples were kept at 4 °C (or −20 °C for a longer period). The same procedure was used for the isolated *F. liguloides* parasites and for non-infected individuals used as negative control. A 1.2% agarose gel was prepared for screening genomic DNA extractions with 4 µL of sample.

### 2.3. Primer Design, PCR Amplification and Sequencing

A pair of primers for amplification and sequencing of 18S rDNA of our cestodes were designed based on ten sequences of hymenolepidids available from GenBank (for primer information, see details in Table S2). Sequences were aligned with MEGA 11 software [40] to identify suitable regions for primer design. We selected a gene fragment including three variable regions (V3, V4, V5) flanked by more conserved ones. The following non-degenerate primers were designed accordingly: forward HymF1 (5′-GATCAATTGGAGGGCAAGT-3′) and reverse HymR1 (5′-CTGTCATGACGGTGATTGA3′). We attempted to amplify a fragment with an expected size of 1462 base pairs (bp) of the 18S gene of cysticercoids of the four genera tested.

A PCR amplification was performed in total volume of 10 µL containing: 1 µL 10× Invitrogen PCR buffer, 1 µL MgCl_2_ (50 mM), 1 µL dNTP (2 mM), 1 µL primer set mix HymF1 + HymR1 (2 µM), 0.1 µL (0.6 U) Taq polymerase (Invitrogen, Carlsbad, CA, USA), 4.9 µL ultrapure water, and 0.5–1 µL DNA template. The PCR consisted of an initial denaturation step at 94 °C for 5 min, followed by 32 cycles (denaturation at 94 °C for 40 s; primer annealing at 54 °C for 40 s; extension at 72 °C for 50 s) and a final extension at 72 °C for 10 min. PCR products were visualized by electrophoresis on 1.5% TAE agarose gel with SYBR green staining under ultraviolet transillumination and using a ladder of 1Kb (Invitrogen) to determine fragment size. DNA re-amplifications were performed using PCR products as templates in a second round of PCR. To do that, each DNA band was excised from the gel with sterile carbon steel, transferred to a 0.2 mL sterilized microtube with 50–70 µL of ultrapure water and kept at 4 °C overnight. PCR for re-amplifications was performed in a volume of 30 µL containing: 3 µL of 10× PCR buffer, 3 µL of 50 mM MgCl_2_, 3 µL of 2 mM dNTP, 3 µL of 2 µM primer set mix, 0.36 µL Taq polymerase (Invitrogen), 16.64 µL ultrapure water, and 1 µL amplified DNA, and selecting the same thermocycling conditions described above. PCR products (2 µL) were loaded on 1.5% TAE agarose gel before sequencing. Amplicons were sent to Macrogen Inc. (Seoul, Republic of Korea) for purification and bi-directional sequencing with the designed Hym-F1/Hym-R1 primers. Amplicons were obtained for a total of 83 samples, but only sequences with the best quality were used for the analyzes. Sixty hymenolepidids (including specimens of the six taxa, Table 1) were successfully sequenced, which meant a success rate of 72.3%. After trimming off the two ends, the size of our sequences ranged from 1113 to 1149 nucleotides. Of the DNA samples that did not amplify (N = 43), 81.4% corresponded to samples collected more than 10 years ago.

### 2.4. Cestode Sequence Analysis and Genetic Variations

Raw nucleotide sequences were assembled and manually edited with GENEIOUS 11.1.5 (Biomatters Ltd., Auckland, New Zealand). The 18S sequences were checked with the Basic Local Alignment Search Tool (BLAST) and the NCBI nucleotide database in general for preliminary molecular identification [41]. We also used other available hymenolepidid sequences from GenBank for our analysis (matching sequences recorded in bird hosts—altogether 9—and 3 selected from mammalian hosts, see Table 1) and the closest available taxon outside of hymenolepidids for an outgroup (i.e., *Dilepis undula*; [29]). Sequences were aligned using the MUSCLE algorithm in MEGA 11 [40]. We assessed nucleotide composition (i.e., the relative frequencies of the four nucleotides) and GC content (%GC) for each taxon with MEGA 11, and the number of polymorphic sites and haplotype diversity in newly generated sequences with DnaSP v. 6. [42]. The most likely evolutionary model was determined based on the corrected Akaike Information Criterion (AICc) in JModelTest v 2.1.10 [43,44]. We chose to use the General Time Reversible (GTR) evolutionary model with a gamma shape parameter (+G) and invariable sites (+I) for construction of Maximum Likelihood (ML) in MEGA 11 and Bayesian inference (BI) in MrBayes [45,46,47] phylogenetic trees as this was the best scoring model (second overall) in JModelTest available in both MEGA 11 and MrBayes version 3.2.7a softwares. ML analyzes were performed with the complete deletion of sites containing gaps and 1000 bootstrap replicates. For BI analyzes, we applied the Markov Chain Monte Carlo (MCMC) method for 1.2 million generations until standard deviation of split frequencies reached <0.01. The trees were sampled every 1000 generations and initial 25% of produced trees were discarded as burn-in. We used FigTree v. 1.4.4 [48] to visualize a single tree.

The pairwise genetic distance (inter- and intragroup) of cestode taxa was calculated with a p-distance and Tamura Nei evolutionary model (TN93; [49]) with a discrete γ model (+G; γ = 1.00) implemented in MEGA 11, both with 1000 bootstrap replicates and complete deletion of sites containing gaps.

## 3. Results

PCR amplification of the genomic DNA yielded partial 18S rDNA sequences for the six cestode taxa assayed, representing four cestode genera.

### 3.1. Genetic Diversity

Comparing the sixty 18S new sequences obtained in this study (Table 1) (1085 unambiguously aligned positions), there were 227 variable sites, of which 225 were parsimoniously informative. The nucleotide composition of sequences had a mean percentage of GC of 49.31%, with slight differences among genera. *Fimbriarioides* sp. had the highest percentage of GC (51.52%), followed by *Confluaria* (50.42%) and *Hymenolepis* (50.0%), whereas the genus *Flamingolepis* exhibited the lowest GC content (between 48.43% and 48.68%).

Levels of nucleotide variation detected between pairs of the hymenolepidid taxa sequenced in this study (Table 1), and nine hymenolepidid taxa found in birds, three randomly chosen species from mammals, and the outgroup *D. undula* available in GenBank (Table 1) are presented in Table S3. In this study, the highest similarity (i.e., lowest genetic distance) was found between *Flamingolepis* and between *Confluaria podicipina* and *Hymenolepis californicus*, while *Flamingolepis liguloides* and *Fimbriarioides* were the most divergent (Table 2).

**Figure 1:**
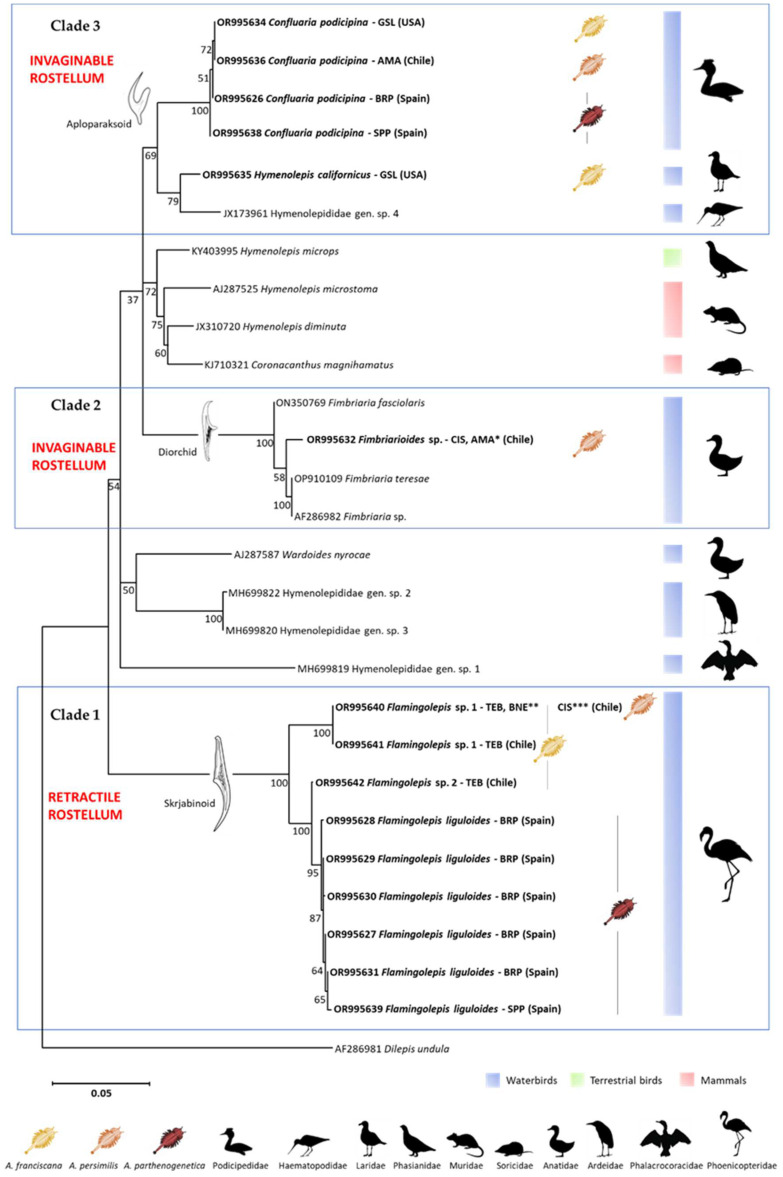
Maximum likelihood (ML) phylogenetic tree based on partial 18S rDNA sequences of 18 hymenolepidid taxa. GenBank accession numbers are shown. Identical sequences recorded at different localities are indicated with asterisks (* OR995637, ** OR995625, *** OR995633). Sequences generated in this study are marked in bold. *Dilepis undula* (family: Dilepididae) was used as outgroup. The three major clades revealed by the present study, including host associations and rostellum morphology, are indicated. Numbers on branches are bootstrap values. The scale bar shows the number of substitutions per site.

The interspecific differences considerably exceeded intraspecific variation (Table 2 and Table 3). Except for *Flamingolepis* sp. 1 in TEB lagoon and *F. liguloides* in BRP, for which genetic variability was detected (two and five haplotypes, respectively; Table 1), no variable positions in the sequences for the remaining cestode taxa were observed at intra-population level. Intraspecific genetic variation was observed for *C. podicipina*, *F. liguloides*, and *Flamingolepis* sp. 1 (Table 3, Figure 1). There was no indication of the existence of cryptic species (Table 2).

### 3.2. Phylogenetic Analyzes

A phylogenetic analysis was performed on a dataset of 27 unique nucleotide sequences of hymenolepidids (15 newly produced haplotypes, 9 sequences previously available in GenBank of bird hymenolepidid parasites, and 3 sequences of mammalian hymenolepidids) and one outgroup sequence (*D. undula*). Maximum likelihood (ML) and Bayesian inference (BI) produced trees with similar topologies, with some differences in primary branches, but for which nodal support was generally below 50% (ML tree shown; Figure 1). Both ML tree (Figure 1) and BI tree (Figure S1) placed newly generated sequences in three separate clusters (1—*Flamingolepis*, 2—*Fimbriarioides*, and 3—*Confluaria podicipina* and *Hymenolepis californicus* clades). Hymenolepidid taxa parasitizing on mammal hosts, together with one taxon found in terrestrial birds (*Hymenolepis microps* found in the Willow ptarmigan *Lagopus lagopus* [50]), belonged to a separate cluster (Figure 1).

All cestode specimens clustered well within their corresponding genera, confirming previous morphological identifications (Figure 1). We found common and local haplotypes, suggesting that both *Artemia* taxon and geographical distance were important. *Confluaria podicipina* was recorded in different *Artemia* species (*A. franciscana*, *A. persimilis*, and *A. parthenogenetica*), but a unique and different haplotype was found in each locality (Table 1, Figure 1). For the *Flamingolepis* sp. 1 associated with South American flamingos, a common haplotype was detected for the two host species (i.e., *A. franciscana* and *A. persimilis*; Table 1), but another haplotype was also found in *A. franciscana* from TEB lagoon in northern Chile. In the case of *Fimbriarioides* sp., recorded in *A. persimilis* in two lagoons from southern Chile (AMA and CIS), a common haplotype was detected at both localities.

## 4. Discussion

Accurate identification of larval cestodes (i.e., cysticercoids) is important but, in certain cases, can be difficult due to their limited morphological characters, scarce knowledge of larval morphology, and unknown life cycles. Molecular techniques can help with identification. In this study, we provided: (1) a cheap and faster procedure for parasite DNA extraction that overcomes the generally complex and time-consuming isolation of microscopic parasites from the host (an *Artemia* hosting a single cysticercoid allowed amplification by PCR); (2) a pair of new primers for the 18S rDNA gene for hymenolepidids; (3) novel molecular data for cestodes of aquatic birds.

The method described had a high rate of amplification success, avoided co-amplification of the host DNA and validated the effectiveness of the primers to amplify cysticercoids of different size, from the smallest (*C. podicipina* 72–147 × 36–89 µm, [10]) to the biggest (*F. liguloides* 560–810 × 372–597 µm, [30]). Our findings highlight the usefulness of DNA data for the identification of parasite larvae, this being a feasible technique for routine analysis in invertebrate intermediate hosts.

### 4.1. Molecular Novelties and the Diversity of Avian Cestodes

Our research is a significant contribution to avian cestodes’ molecular information. A total of eighteen partial 18S rDNA sequences for six taxa of four genera, that as adults parasitize different waterbirds (flamingos, grebes, gulls, and ducks), have been deposited in GenBank [41]. These represent the first molecular data for *Artemia* parasites. Indeed, there were previously no sequences of any gene for the genus *Flamingolepis* or *Fimbriarioides*, and only limited data for other parasite taxa with the potential to infect brine shrimp hosts. For parasites of gulls (Laridae) of the genus *Wardium*, several species use *Artemia* as an intermediate host (i.e., *W. fusa*, *W. stellorae*, and *Wardium* sp., in Asia, Europe and America, respectively; [12]) but only one 28S sequence of *Wardium cirrosa* from polychaetes is available. For the genus *Hymenolepis*, most available data were for mammalian parasites such *H. diminuta* and *H. microstoma* parasitizing rodents, with *H. microps* from *Lagopus lagopus* (Phasianidae) being the only molecular record for avian parasites to date. We provided the first data of *H. californicus*, a parasite associated with Nearctic gulls such as *Larus californicus* [33]. For the genus *Confluaria* (parasite of grebes), there was only one pre-existing 28S sequence for *Confluaria pseudofurcifera* infecting *Podiceps cristatus* [51] and we presented the first data for *C. podicipina*.

Our study offered molecular data for cestodes from different geographical regions and continents, with some species recorded in several brine shrimp and avian hosts species. For example, *C. podicipina* is the most cosmopolitan taxon with records in *A. parthenogenetica* from Europe, *A. franciscana* from North America and *A. persimilis* from South America, which can be associated with different grebe species acting as potential final hosts in each locality (e.g., *Podiceps nigricollis*, *P. auritus*, and *P. occipitalis*; Table 1), existing direct evidences confirming that the life history of this hymenolepidid occurs between brine shrimp (intermediate host) and the silvery grebe *P. occipitalis* (final host) [52]. The flamingo parasite *Flamingolepis* sp. 1 was recorded in both *A. franciscana* and *A. persimilis* from Chile, while *Fimbriarioides* sp. was exclusively infecting *A. persimilis* in southern Chile. Studies like this can provide key information about host–parasite associations, geographical distribution, and life cycles of parasites.

Morphology has been the standard method for cestode larvae identification, but it usually results in provisional identifications pending complementary information from adult specimens or genetic confirmation. The scarce knowledge of larval morphology and life cycles of avian cestodes are important limitations when using comparative morphology for parasite identification. Molecular research can help to clarify the taxonomy of poorly studied genera such *Flamingolepis* Spasskii et Spasskaya, 1954. The differences we found among the sequences of *Flamingolepis* spp. indicated: (1) the Chilean species (*Flamingolepis* sp. 1 and sp. 2) and the European (*F. liguloides*) were closely related to each other (clade *Flamingolepis*) and distantly related to the other three genera analyzed; (2) the cysticercoids of *Flamingolepis* sp. 1 belonged to a different species (as already suggested based on morphology, [10,12]) since they clustered in a separate sub-clade (see Figure 1 and Figure S1). However, this was less likely for *Flamingolepis* sp. 2, probably due to a single analyzed specimen providing insufficient molecular data. From now on, we recommend genetic identification as the fastest method for a preliminary diagnosis, but morphological and molecular evidence should be combined for a better classification of larval specimens.

### 4.2. Genetic Variation of Hymenolepidids in Relation to Aquatic Birds

Understanding the genetic diversity of parasites in wild fauna is important for disease control, elucidating patterns of transmission, and biodiversity conservation. The differences detected in hymenolepidid sequences may be related to final host dynamics and parasite specificity. For *C. podicipina*, interpopulation variations may reflect a geographical pattern associated with the ecology of grebes acting as final hosts in each region. A closer relationship between the American populations suggests a role of migratory movements of grebes in shaping this pattern, and greater isolation between Old and New World populations. The similarity within Spanish populations may be related to the small number of sequences, but also with local selective pressures affecting parasite reproduction (such as a shortage of avian hosts in certain years). However, further studies are needed for a better understanding of the role of host species in the biogeography of this cestode. The *Flamingolepis* genus exhibited a higher level of nucleotide variability, particularly the species parasitizing European flamingos (i.e., *F. liguloides*). It is noteworthy that there was reduced variability for the congener *Flamingolepis* sp. 1, despite it being detected in both *A. franciscana* and *A. persimilis* inhabiting contrasting and distant habitats in Chile [53]. Spatio-temporal movements of the Chilean flamingo potential final hosts [35,54] could explain the similarity of the sequences, as well as the infection seasonality found in brine shrimps [53]. Since long-distance movements of flamingos are common, genetic variation between populations of geographically distant flamingo parasites was not expected. The lack of sequence variation for *Fimbriarioides*, recorded in both Patagonian lagoons and morphologically identified as the same taxa [10,53], might be explained in a similar way. Anatids are known to disperse aquatic organisms (such as invertebrates and plant seeds) between breeding and wintering grounds during north–south migrations [55]. Anatids (such as shelducks or dabbling ducks) acting as potential final hosts of *Fimbriarioides* sp. [10] can easily move between the two lagoons (c. 350 km), likely dispersing their parasites, as has been suggested in other crustacean host–parasite systems, such *Daphnia*-microparasites [56]. Using less conservative genetic markers, parasite dispersal by waterbirds could be further explored.

### 4.3. Congruence between Molecular Data, Larvae Morphology and Associations with Avian Final Hosts: Some Notes on Phylogeny

Previous studies showed that the rostellar apparatus and strobila of adult parasites are useful characters for explaining phylogenetic relationships among mammalian hymenolepidids [28,29]. Our results revealed a strong congruence between molecular identification and larvae morphology (for morphological descriptions of the parasites see [10,12,30,33,39]) and demonstrated: (i) a close relationship between the two genera with 10 aploparaksoid hooks and invaginable rostellum (i.e., *C. podicipina* and *H. californicus*); (ii) likewise, between *Fimbriarioides* sp. and *Fimbriaria* spp., both with 10 diorchid hooks and invaginable rostellum; (iii) a distant *Flamingolepis* clade which clustered all taxa with eight skrjabinoid hooks and retractile rostellum (i.e., *Flamingolepis* spp.) (Figure 1). Therefore, larval morphology (particularly related to the rostellum) has a significant value explaining interrelationships, but other factors, such as the ecology of final hosts, parasite specificity and life cycles, could also explain phylogenetic relationships within this family. For instance, *Flamingolepis* spp. are parasites of flamingos (Phoenicopteridae-Phoenicopteriformes) in the Nearctic and Palaearctic, [10,12,30]. The genus *Fimbriarioides* has been recorded from Anatidae, with the species *F. tadornae*, a parasite of the common shelduck *Tadorna tadorna*, as the only known species infecting *Artemia* in the Palaearctic [39]. Phylogenetic trees showed that *Fimbriarioides* sp. from Chile was closely related to *Fimbriaria* spp. found in the Mallard *Anas platyrhynchos* in the USA (*Fimbriaria* sp., [57]) and Ukraine (*Fimbriaria fasciolaris* and *F. teresae*, [58]), all having Anatidae as final hosts. *H. californicus* (parasite of gulls, Laridae-Charadriiformes) was closely related to a parasite found in the variable oystercatcher *Haemotopus unicolor* (*Hymenolepididae* gen. sp. 4; [59]), with both having Charadriiform birds as final hosts. *C. podicipina*, the parasite of grebes (Podicipedidae-Podicipediformes) was in a separate clade.

### 4.4. Future Directions

Molecular studies are useful for discovering new parasite species, contributing to biodiversity [6,56,60], and for advances on waterbird parasites and their life cycles [51,61]. This is particularly relevant for protected waterbirds for which the knowledge of their parasite fauna is little known, such as the case of flamingos in Chile (present study). Furthermore, considering the diversity of cestodes recorded in brine shrimps (22 species from 10 genera and 3 families [12,37]), and their cosmopolitan distribution, they are an excellent model for exploring genetic diversity and biogeographical patterns of avian parasites. Their sequences can be used for developing taxon-specific primers as a diagnostic tool for multiple infections.

Now that these cestode taxa have been characterized with 18S, it is feasible to further investigate their life cycles and distribution by the analysis of eDNA in the feces from potential avian hosts, as conducted for other parasites (e.g., [7,62]). Moreover, very little is known about the cestode dispersal by waterbirds across regions and continents, and even less between hemispheres. Molecular studies on parasites from invertebrate hosts and avian fecal samples collected at the same localities and seasons will improve our understanding of bird-mediated dispersal in shaping parasite genetic patterns, and ultimately predict parasite transmission pathways.

Finally, molecular identification of waterbird parasites and genetic diversity will contribute to a more integrated and comparative analysis (local vs. regional) of parasite–host evolutionary dynamics and of the ecosystem services provided by hypersaline wetlands, which is important considering their disappearance and degradation around the world [3,63].

## 5. Conclusions

Molecular studies on cestodes, with a special focus on the characterization of avian parasites, are required to advance the knowledge of cestode biodiversity and phylogeny. Our molecular approach using infected crustaceans, and avoiding invasive techniques in vertebrates, is helpful in this sense. Our study provides valuable data that help to resolve the phylogenetic history of avian hymenolepidids. However, further research, extending both the number of taxa and genes, is required to obtain a comprehensive understanding of phylogenetic relationships within hymenolepidids, and to elucidate interrelationships among other cestode families. Future sequences from adult parasite specimens would also improve our understanding of the specificity and biogeography of these parasites.

## Figures and Tables

**Table 1:** Cestode taxa included in the genetic distance and phylogenetic analysis. Columns show the intermediate host, geographic origin, length of 18S fragment, haplotypes, sequences access number, and final host. Sequences obtained in this study are indicated in bold. Final hosts are also indicated as G, grebes; F, flamingos; D, ducks; S, shorebirds; WB, other waterbirds; B, other birds; M, mammals.

Cestode Taxa	Intermediate Host	Geographic Origin	Size (bp)	Haplotype (no. Sequences)	Acc. no. GenBank	Final Host
*Confluaria podicipina*	*Artemia* *franciscana*	Great Salt Lake, Utah, USA (GSL)	1115	1 (8)	**OR995634**	G: *Podiceps auratus* *, *P. nigricollis* *
*Confluaria podicipina*	*Artemia* *persimilis*	Amarga lagoon, Chile (AMA)	1115	2 (2)	**OR995636**	G: *Podiceps occipitalis* *
*Confluaria podicipina*	*Artemia* *parthenogenetica*	San Pedro del Pinatar, Murcia, Spain (SPP)	1116	3 (2)	**OR995638**	G: *Podiceps nigricollis* *
*Confluaria podicipina*	*Artemia* *parthenogenetica*	Bras del Port salterns, Alicante, Spain (BRP)	1115	4 (1)	**OR995626**	G: *Podiceps nigricollis* *
*Fimbriaria fasciolaris*	NA	Chernihiv Oblast’, Ukraine	2039		ON350769	D: *Anas platyrhynchos*
*Fimbriaria* sp.	NA	Lake Butte des Morts, Wisconsin, USA	2137		AF286982	D: *Anas platyrhynchos*
*Fimbriaria teresae*	NA	Chernihiv Oblast’, Ukraine	2020		OP910109	D: *Anas platyrhynchos*
*Fimbriarioides* sp.	*Artemia persimilis*	Amarga lagoon, Chile (AMA)	1149	1 (2)	**OR995637**	D: Anatidae
*Fimbriarioides* sp.	*Artemia persimilis*	Los Cisnes lagoon, Chile (CIS)	1149	1(4)	**OR995632**	D: Anatidae
*Flamingolepis* *liguloides*	*Artemia* *parthenogenetica*	Bras del Port salterns, Alicante, Spain (BRP)	1113	1 (1) 2 (1) 3 (2) 4 (1) 5 (1)	**OR995627** **OR995628** **OR995629** **OR995630** **OR995631**	F: *Phoenicopterus roseus*
*Flamingolepis* *liguloides*	*Artemia* *parthenogenetica*	San Pedro del Pinatar salterns, Murcia, Spain (SPP)	1113	6 (1)	**OR995639**	F: Phoenicopterus roseus *
*Flamingolepis* sp. 1	*Artemia* *persimilis*	Los Cisnes lagoon, Chile (CIS)	1117	1 (14)	**OR995633**	F: Phoenicopterus chilensis *
*Flamingolepis* sp. 1	*Artemia* *franciscana*	Tebenquiche lagoon, Chile (TEB)	1117	1 (7) 2 (1)	**OR995640** **OR995641**	F: *Phoenicopterus* chilensis *
*Flamingolepis* sp. 1	*Artemia* *franciscana*	Barros negros lagoon, Chile (BNE)	1117	1 (9)	**OR995625**	F: *Phoenicopterus* chilensis *
*Flamingolepis* sp. 2	*Artemia* *franciscana*	Tebenquiche lagoon, Chile (TEB)	1113	1 (1)	**OR995642**	F: *Phoenicopterus* chilensis *, Phoenicoparrus spp. *
Hymenolepididae gen. sp. 1	NA	Ciénega de Santa Clara, Sonora, Mexico	2076		MH699819	WB: *Nannopterum* *auritus*
Hymenolepididae gen. sp. 2	NA	Los Chivos, Veracruz, Mexico	2016		MH699822	WB: *Nycticorax* *nycticorax*
Hymenolepididae gen. sp. 3	NA	Lago de Catemaco Veracruz, Mexico	2042		MH699820	WB: *Nyctanassa violacea*
Hymenolepididae gen. sp. 4	NA	Tasman Bay, New Zealand	1971		JX173961	S: *Haematopus unicolor*
*Hymenolepis* *californicus*	*Artemia* *franciscana*	Great Salt Lake, Utah, USA (GSL)	1118	1 (2)	**OR995635**	S: *Larus californicus*, *L. dalawarensis*
*Hymenolepis microps*	*Lagopoecus affinis* *, *Goniodes lagopi* *	Kattfjord, Troms County, Norway	2233		KY403995	B: *Lagopus lagopus*
*Wardoides nyrocae*	NA	NA	2186		AJ287587	WB: *Cygnus olor*
*Coronacanthus magnihamatus*	NA	Boyana River, Bulgaria	1996		KJ710321	M: *Neomys fodiens*
*Hymenolepis diminuta*	NA	Lab strain	2148		AF286983	M: *Rattus norvegicus*
*Hymenolepis* *microstoma*	NA	Lab strain	2140		AJ287525	M: Mus sp.
*Dilepis undula*	NA	Wimbourne, Dorset, UK	2091		AF286981	B: *Turdus merula*

* Indicates putative host species.

**Table 2:** Estimates of evolutionary divergence between hymenolepidid taxa sequenced in this study. The number of base differences per site from averaging over all sequence pairs between groups of sequences are shown below the diagonal. Standard error estimate(s) are shown above the diagonal and were obtained by a bootstrap procedure (1000 replicates). The analysis involved 60 nucleotide sequences. All positions containing gaps and missing data were eliminated (complete deletion option). There was a total of 1085 positions in the final dataset. The analysis was conducted in MEGA11 ([40]).

Cestode Taxa	1	2	3	4	5	6
1 *Flamingolepis* sp. 1		0.007	0.007	0.011	0.010	0.010
2 *Flamingolepis* sp. 2	0.055		0.002	0.010	0.010	0.010
3 *Flamingolepis liguloides*	0.059	0.007		0.011	0.010	0.010
4 *Fimbriarioides* sp.	0.147	0.143	0.147		0.010	0.010
5 *Confluaria podicipina*	0.132	0.127	0.130	0.110		0.007
6 *Hymenolepis californicus*	0.139	0.135	0.142	0.116	0.061	

**Table 3:** Estimates of mean genetic divergence over sequence pairs within hymenolepidid taxa sequenced in this study. The number of base differences per site from averaging over all sequence pairs within each group are shown. This analysis involved 60 nucleotide sequences. All positions containing gaps and missing data were eliminated (complete deletion option). There were a total of 1085 positions in the final dataset. The presence of n/c in the results denotes cases in which it was not possible to estimate evolutionary distances.

Cestode Taxa	Mean ± Standard Error
*Confluaria podicipina*	0.0012 ± 0.0006
*Flamingolepis* sp. 1	0.0001 ± 0.0001
*Flamingolepis* sp. 2	n/c
*Flamingolepis liguloides*	0.0019 ± 0.0008
*Fimbriarioides* sp.	0.0000 ± 0.0000
*Hymenolepis californicus*	0.0000 ± 0.0000

## Data Availability

Data generated and analyzed during this study are contained within the article and its Supplementary Materials. All new partial 18S sequences are accessible in GenBank repository. Samples ID (accession numbers) are also provided in Table 1 and Figure 1.

## Supplementary Materials

The following supporting information can be downloaded at: https://www.mdpi.com/article/10.3390/ani14030397/s1, Table S1: List of brine shrimp individuals analyzed; Table S2: Sequences used for primer design [29,50,57,59,64,65,66]; Table S3: Estimates of genetic divergence over sequence pairs between cestode taxa; Figure S1: Bayesian Inference (BI) tree showing phylogenetic relationships among hymenolepidid taxa.

## Appendix A

### Description of the DNA Extraction Method for Our Material

Artemia samples (individual or pools) were homogenized in 300 µL of Cell Lysis Solution (Wizard®, Promega, Madison, Wi, USA) using an ultrasonic homogenizer (Omni Sonic Ruptor 400, Omni International Inc., Kennesaw, GE, USA) and 10 pulses of 2 s (step 1). Then, we added 200 µL of buffer for a second homogenization, applying 5 pulses of 2 s (step 2). This process was done over ice to avoid overheating of the sample. Once the animal tissue appeared disaggregated, 10 µL of RNAse solution (10 mg/mL, Promega, Madison, WI, USA) was added to the sample and incubated for 2 h at 37 °C followed by 1 h at 56 °C with the addition of 10 µL of proteinase K (10 mg/mL, Merck KGaA, Darmstadt, Germany). After that, 300 µL of phenol was added to each tube, mixed carefully by hand and centrifuged at 11,000 rpm for 15 min with an Eppendorf 5804R centrifuge. The supernatant was transferred to another tube with 300 µL of chloroform: alcohol isoamilic (24:1), mixed and centrifuged for 10 min to 11,000 rpm. The supernatant was transferred to a new tube at which sodium acetate (NaAc, 3M) (10% of the total sample volume) and isopropanol (0.7 volumes) were added. After keeping the tubes 30 min at 4 °C, they were centrifuged at 11,000 rpm for 20 min and the supernatant was poured off. The DNA pellet was washed with 200 µL of 70% ethanol and centrifuged at 11,000 rpm for 10 min. The DNA was dried at 40 °C and suspended in 50 µL of molecular biology grade water (Qiagen, Hilden, Germany) until used.

## References

[1] Marcogliese D.J. (2004). Parasites: Small players with crucial roles in the ecological theater. EcoHealth.

[2] Mariaux J., Tkach V.V., Vasileva G.P., Waeschenbach A., Beveridge I., Dimitrova Y.D., Haukisalmi V., Greiman S.E., Littlewood D.T.J., Makarikov A.A., Caira J.N., Jensen K. (2017). Cyclophyllidea van Beneden in Braun, 1900. Planetary Biodiversity Inventory (2008–2017): Tapeworms from Vertebrate Bowels of the Earth.

[3] Gajardo G., Redón S. (2019). Andean hypersaline lakes in the Atacama Desert, northern Chile: Between lithium exploitation and unique biodiversity conservation. Conserv. Sci. Pract..

[4] Gutiérrez J.S., Moore J.N., Donnelly J.P., Dorador C., Navedo J.G., Senner N.R. (2022). Climate change and lithium mining influence flamingo abundance in the Lithium Triangle. Proc. R. Soc. B.

[5] Criscione C.D., Poulin R., Blouin M.S. (2005). Molecular ecology of parasites: Elucidating ecological and microevolutionary processes. Mol. Ecol..

[6] Poulin R., Morand S. (2005). Parasite Biodiversity.

[7] Briscoe A.G., Nichols S., Hartikainen H., Knipe H., Foster R., Green A.J., Okamura B., Bass D. (2022). High-throughput sequencing of faeces provides evidence for dispersal of parasites and pathogens by migratory waterbirds. Mol. Ecol. Res..

[8] Douchet P., Boissier J., Mulero S., Ferté H., Doberva M., Allienne J.F., Toulza E., Bethune K., Rey O. (2022). Make visible the invisible: Optimized development of an environmental DNA metabarcoding tool for the characterization of trematode parasitic communities. Environ. DNA.

[9] Poulin R., Hay E., Jorge F. (2019). Taxonomic and geographic bias in the genetic study of helminth parasites. Int. J. Parasitol..

[10] Redón S., Vasileva G.P., Georgiev B.B., Gajardo G. (2019). First report of cestode infection in the crustacean *Artemia persimilis* from Southern Chilean Patagonia and its relation with the Neotropical aquatic birds. PeerJ.

[11] Babero B., Cattan P., Jensen L. (1981). Helmintofauna de Chile. IX. *Flamingolepis chileno* sp. n. parásito de *Phoenicoparrus andinus* Philippi (Cestoda: Hymenolepididae). Bol. Mus. Nac. Hist. Nat. Chile.

[12] Redón S., Vasileva G.P., Georgiev B.B., Gajardo G. (2020). Exploring parasites in extreme environments of high conservational importance: *Artemia franciscana* (Crustacea: Branchiopoda) as intermediate host of avian cestodes in Andean hypersaline lagoons from Salar de Atacama, Chile. Parasitol. Res..

[13] Formenti N., Chiari M., Trogu T., Gaffuri A., Garbarino C., Boniotti M.B., Corradini C., Lanfranchi P., Ferrari N. (2018). Molecular identification of cryptic cysticercosis: *Taenia ovis krabbei* in wild intermediate and domestic definitive hosts. J. Helminthol..

[14] Cháves-González L.E., Morales-Calvo F., Mora J., Solano-Barquero A., Verocai G.G., Rojas A. (2022). What lies behind the curtain: Cryptic diversity in helminth parasites of human and veterinary importance. Curr. Res. Parasitol. Vector-Borne Dis..

[15] Greben O., Kudlai O., Kornyushin V.V. (2019). The intermediate hosts of *Wardium cirrosa* (Krabbe, 1869) Spassky, 1961 (Cestoda, Cyclophyllidea, Aploparaksidae) in Ukraine. Parasitol. Res..

[16] Myczka A.W., Jeżewski W., Filip–Hutsch K.J., Pyziel A.M., Kowal J., Demiaszkiewicz A.W., Laskowski Z. (2020). The morphological and molecular identification of the tapeworm, *Taenia lynciscapreoli*, in intermediate and definitive hosts in Poland. Int. J. Parasitol. Parasites Wildl..

[17] Mariaux J. (1998). A molecular phylogeny of the Eucestoda. J. Parasitol..

[18] Olson P.D., Ruhnke T.R., Sanney J., Hudson T. (1999). Evidence for host-specific clades of tetraphyllidean tapeworms (Platyhelminthes: Eucestoda) revealed by analysis of 18S ssrDNA. Int. J. Parasitol..

[19] Sharma S., Lyngdoh D., Roy B., Tandon V. (2016). Molecular phylogeny of Cyclophyllidea (Cestoda: Eucestoda): An in-silico analysis based on mtCOI gene. Parasitol. Res..

[20] Foronda P., Casanova J.C., Martinez E., Valladares B., Feliu C. (2005). *Taenia* spp.: 18S rDNA microsatellites for molecular systematic diagnosis. J. Helminthol..

[21] McManus D.P. (2006). Molecular discrimination of taeniid cestodes. Parasitol. Int..

[22] Al-Amri O., Al-Quraishy S., Al-Shaebi E.M., Aljawdah H., Abdel-Gaber R. (2022). Molecular identification of the rodent-borne pathogen *Rodentolepis nana* using the genetic markers of ITS-1, 18S, and 28S rDNA. Mol. Biol. Rep..

[23] Šnábel V., Kuzmina T.A., Antipov A.A., Yemets O.M., Cavallero S., Miterpáková M., D’Amelio S., Antolová D., Vasilkova Z., Sałamatin R. (2022). Molecular study of *Echinococcus granulosus* cestodes in Ukraine and the first genetic identification of *Echinococcus granulosus sensu stricto* (G1 genotype) in the country. Acta Parasitol..

[24] Haukisalmi V., Hardman L.M., Foronda P., Feliu C., Laakkonen J., Niemimaa J., Lehtonen J.T., Henttonen H. (2010). Systematic relationships of hymenolepidid cestodes of rodents and shrews inferred from sequences of 28S ribosomal RNA. Zool. Scr..

[25] Greiman S.E., Tkach V.V., Cook J.A. (2013). Description and molecular differentiation of a new *Staphylocystoides* (Cyclophyllidea: Hymenolepididae) from the dusky shrew *Sorex monticolus* in Southeast Alaska. J. Parasitol..

[26] Makarikov A.A., Mel’nikova Y.A., Tkach V.V. (2015). Description and phylogenetic affinities of two new species of *Nomadolepis* (Eucestoda, Hymenolepididae) from Eastern Palearctic. Parasitol. Int..

[27] Tkach V.V., Kinsella J.M., Greiman S.E. (2018). Two new species of *Staphylocystoides* Yamaguti, 1959 (Cyclophyllidea: Hymenolepididae) from the masked shrew *Sorex cinereus* in North America. J. Parasitol..

[28] Neov B., Vasileva G.P., Radoslavov G., Hristov P., Littlewood D.T.J., Georgiev B.B. (2019). Phylogeny of hymenolepidid cestodes (Cestoda: Cyclophyllidea) from mammalian hosts based on partial 28S rDNA, with focus on parasites from shrews. Parasitol. Res..

[29] Neov B., Vasileva G.P., Radoslavov G., Hristov P., Littlewood D.T.J., Georgiev B.B. (2021). Phylogeny of hymenolepidids (Cestoda: Cyclophyllidea) from mammals: Sequences of 18S rRNA and COI genes confirm major clades revealed by the 28S rRNA analyses. J. Helminthol..

[30] Georgiev B.B., Sánchez M.I., Green A.J., Nikolov P.N., Vasileva G.P., Mavrodieva R.S. (2005). Cestodes from *Artemia parthenogenetica* (Crustacea, Branchiopoda) in the Odiel Marshes, Spain: A systematic survey of cysticercoids. Acta Parasitol..

[31] Georgiev B.B., Sánchez M.I., Vasileva G.P., Nikolov P.N., Green A.J. (2007). Cestode parasitism in invasive and native brine shrimps (*Artemia* spp.) as a possible factor promoting the rapid invasion of *A. franciscana* in the Mediterranean region. Parasitol. Res..

[32] Redón S., Amat F., Hontoria F., Vasileva G.P., Nikolov P.N., Georgiev B.B. (2011). Participation of metanauplii and juvenile individuals of *Artemia parthenogenetica* (Branchiopoda) in the circulation of avian cestodes. Parasitol. Res..

[33] Redón S., Berthelemy N.J., Mutafchiev Y., Amat F., Georgiev B.B., Vasileva G.P. (2015). Helminth parasites of *Artemia franciscana* (Crustacea: Branchiopoda) in the Great Salt Lake, Utah: First data from the native range of this invader of European wetlands. Folia Parasitol..

[34] Sánchez M.I., Nikolov P.N., Georgieva D.D., Georgiev B.B., Vasileva G.P., Pankov P., Paracuellos M., Lafferty K.D., Green A.J. (2013). High prevalence of cestodes in *Artemia* spp. throughout the annual cycle: Relationship with abundance of avian final hosts. Parasitol. Res..

[35] Caziani S.M., Olivio O.R., Ramirez E.R., Romano M., Derlindati E.J., Tálamo A., Ricalde D., Quiroga C., Contreras J.P., Valqui M. (2007). Seasonal distribution, abundance, and nesting of Puna, Andean, and Chilean Flamingos. Condor.

[36] Gajardo G., Redón S., Manning A.J. (2019). Hypersaline Lagoons from Chile, the Southern Edge of the World. Lagoon Environments Around the World—A Scientific Perspective.

[37] Kornyychuck Y., Anufriieva E., Shadrin N. (2023). Diversity of parasitic animals in hypersaline waters: A review. Diversity.

[38] Redón S., Amat F., Georgiev B.B., Vasileva G.P., Nikolov P.N., Lafferty K.D., Sánchez M.I., Green A.J. (2023).

[39] Vasileva G., Redón S., Amat F., Nikolov P., Sánchez M., Lenormand T., Georgiev B. (2009). Records of cysticercoids of *Fimbriarioides tadornae* Maksimova, 1976 and *Branchiopodataenia gvozdevi* (Maksimova, 1988) (Cyclophyllidea, Hymenolepididae) from brine shrimps at the Mediterranean coasts of Spain and France, with a key to cestodes from *Artemia* spp. from the Western Mediterranean. Acta Parasitol..

[40] Tamura K., Stecher G., Kumar S. (2021). MEGA11: Molecular Evolutionary Genetics Analysis version 11. Mol. Biol. Evol..

[41] National Center for Biotechnology Information of USA (NCBI). https://www.ncbi.nlm.nih.gov/.

[42] Rozas J., Ferrer-Mata A., Sánchez-DelBarrio J.C., Guirao-Rico S., Librado P., Ramos-Onsins S.E., Sánchez-Gracia A. (2017). DnaSP 6: DNA sequence polymorphism analysis of large data sets. Mol. Biol. Evol..

[43] Darriba D., Taboada G.L., Doallo R., Posada D. (2012). jModelTest 2: More models, new heuristics and parallel computing. Nat. Methods.

[44] Guindon S., Gascuel O. (2003). A simple, fast and accurate method to estimate large phylogenies by maximum-likelihood. Syst. Biol..

[45] Huelsenbeck J.P., Ronquist F. (2001). MRBAYES: Bayesian inference of phylogenetic trees. Bioinformatics.

[46] Ronquist F., Huelsenbeck J.P. (2003). MrBayes 3: Bayesian phylogenetic inference under mixed models. Bioinformatics.

[47] Ronquist F., Teslenko M., van der Mark P., Ayres D.L., Darling A., Höhna S., Larget B., Liu L., Suchard M.A., Huelsenbeck J.P. (2012). MrBayes 3.2: Efficient Bayesian phylogenetic inference and model choice across a large model space. Syst. Biol..

[48] Fig. Tree v. 1.4.4. https://github.com/rambaut/figtree/releases.

[49] Tamura K., Nei M. (1993). Estimation of the number of nucleotide substitutions in the control region of mitochondrial DNA in humans and chimpanzees. Mol. Biol. Evol..

[50] Pistone D., Lindgren M., Holmstad P., Ellingsen N.K., Kongshaug H., Nilsen F., Skorping A. (2018). The role of chewing lice (Phthiraptera: Philopteridae) as intermediate hosts in the transmission of *Hymenolepis microps* (Cestoda: Cyclophyllidea) from the willow ptarmigan *Lagopus lagopus* (Aves: Tetraonidae). J. Helminth..

[51] Presswell B., Bennett J. (2022). Gastrointestinal helminth parasites of the threatened Australasian crested grebe (*Podiceps cristatus australis*, Gould 1844) in New Zealand, with descriptions of *Baruscapillaria kamanae* n. sp. (Nematoda: Trichuridae) and *Cryptocotyle micromorpha* n. sp. (Trematoda: Opisthorchiidae). Syst. Parasitol..

[52] Guagliardo S.E., Graff M.E., Gigola G., Tanzola R.D. (2020). Biological aspects of the life history of *Confluaria podicipina* (Cestoda Hymenolepididae) from a hypersaline pampasic lagoon. Pan-Am. J. Aquat. Sci..

[53] Redón S., Gajardo G., Vasileva G.P., Sánchez M.I., Green A.J. (2021). Explaining variation in abundance and species diversity of avian cestodes in brine shrimps in the Salar de Atacama and other Chilean wetlands. Water.

[54] Mascitti V.L., Bonaventura S.M. (2002). Patterns of abundance, distribution and habitat use of flamingos in the high Andes, South America. Waterbirds.

[55] Green A.J., Lovas-Kiss Á., Reynolds C., Sebastián-González E., Silva G.G., van Leeuwen C.H.A., Wilkinson D.M. (2023). Dispersal of aquatic and terrestrial organisms by waterbirds: A review of current knowledge and future priorities. Freshw. Biol..

[56] Wolinska J., Giessler S., Koerner H. (2009). Molecular identification and hidden diversity of novel *Daphnia* parasites from European lakes. Appl. Environ. Microbiol..

[57] Olson P.D., Littlewood D.T.J., Bray R.A., Mariaux J. (2001). Interrelationships and evolution of the tapeworms (Platyhelminthes: Cestoda). Mol. Phylogenet. Evol..

[58] Greben O., Kornyushin V., du Preez L., Melnychuk V., Yevstafieva V., Syrota Y. (2023). Cestodes of the genus *Fimbriaria* Froelich, 1802 (Cestoda, Cyclophyllidea, Fimbriariinae) from Ukraine, with a key to species level. Acta Parasitol..

[59] Presswell B., Melville D.S., Randhawa H.S. (2012). Tapeworm bolus expelled from New Zealand variable oystercatchers (*Haematopus unicolor*) during handling: First record of this phenomenon in wild birds, and a global checklist of *Haematopus* cestode parasites. Parasitol. Res..

[60] Brooks D.R., Hoberg E.P. (2000). Triage for the biosphere: The need and rationale for taxonomic inventories and phylogenetic studies of parasites. Comp. Parasitol..

[61] Hou Z., Han L., Sun Y., Shen D., Peng Z., Wang L., Zhai Q., Zhou Y., Lu Y., Teng L. (2020). Morphological and phylogenetical analysis reveals that a new tapeworm species (Cestoda: Hymenolepididae) from whooper swan belongs to *Cloacotaenia* not *Hymenolepis*. J. For. Res..

[62] Hartikainen H., Bass D., Briscoe A.G., Knipe H., Green A.J., Okamura B. (2016). Assessing myxozoan presence and diversity using environmental DNA. Int. J. Parasitol..

[63] Wurtsbaugh W.A., Miller C., Null S.E., DeRose R.J., Wilcock P., Hahnenberger M., Howe F., Moore J. (2017). Decline of the world’s saline lakes. Nat. Geosci..

[64] Olson P.D., Caira J.N. (1999). Evolution of the major lineages of tapeworms (Platyhelminthes: Cestoidea) inferred from 18S ribosomal DNA and elongation factor-1α. J. Parasitol..

[65] Littlewood D.T.J., Olson P.D., Littlewood D.T.J., Bray R.A. (2001). Small subunit rDNA and the Platyhelminthes: Signal, noise, conflict, and compromise. Interrelationships of the Platyhelminthes.

[66] Olson P.D., Yoder K., Fajardo LG L.F., Marty A.M., van de Pas S., Olivier C., Relman D.A. (2003). Lethal invasive cestodiasis in immunosuppressed patients. J. Infect. Dis..

