# Supplementary material for: Phylogenetic Relationships of Avian Cestodes from Brine Shrimp and Congruence with Larval Morphology
Source: Animals (Basel). 2024 Jan 25;14(3):397. doi: 10.3390/ani14030397 (PMC10854740; doi:10.3390/ani14030397)
Supplement: Supplementary file 1 [file animals-14-00397-s001.zip › Figure S1_BI phylogenetic tree.pdf]

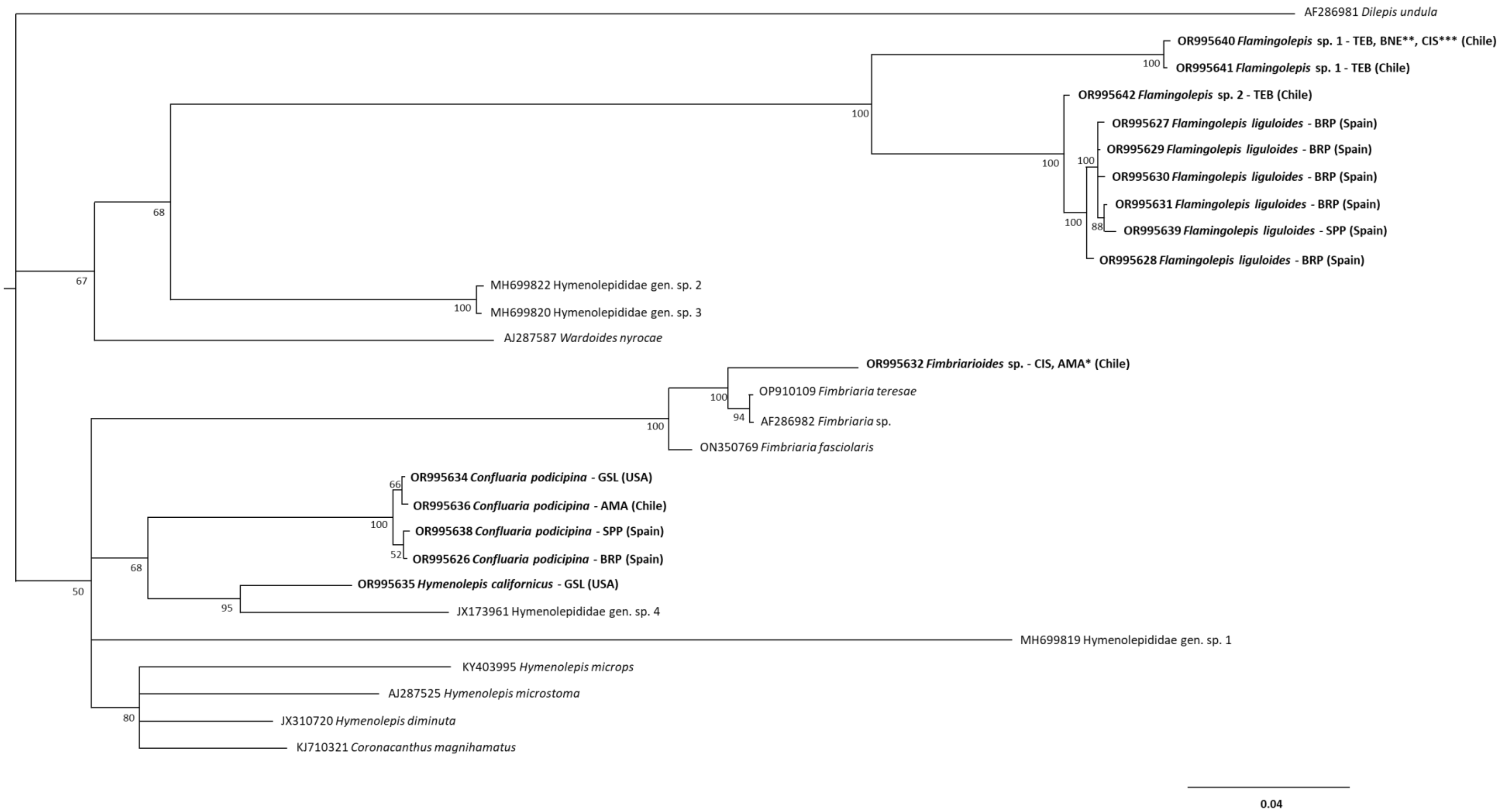

Figure S1. Bayesian inference (BI) phylogenetic tree based on partial 18S rDNA sequences of 18 hymenolepidid taxa. GenBank accession numbers are shown. Identical sequences recorded at different localities are indicated with asterisks (*OR995637, **OR995625, ***OR995633). *Dilepis undula* (family: Dilepididae) is used as outgroup. Newly sequences generated in this study are marked in bold. Numbers on branches are posterior probabilities. Scale bar shows the number of substitutions per site.
