# Supplementary material for: Phylogenetic Relationships of Avian Cestodes from Brine Shrimp and Congruence with Larval Morphology
Source: Animals (Basel). 2024 Jan 25;14(3):397. doi: 10.3390/ani14030397 (PMC10854740; doi:10.3390/ani14030397)
Supplement: Supplementary file 1 [file animals-14-00397-s001.zip › Table S1_List of infected Artemia individuals.pdf]

Table S1. List of infected brine shrimp individuals analysed in the present study indicating *Artemia* species, locality (see abbreviations in the text), collection date, and infective status.

No. individual (brine shrimp)	*Artemia* population	Locality	Country	Collection date	Infective status	
					Intensity	Cestode taxa
1	*Artemia persimilis*	CIS	CHILE	29/11/2017	1	*Flamingolepis* sp. 1
2	*Artemia persimilis*	CIS	CHILE	29/11/2017	1	*Flamingolepis* sp. 1
3	*Artemia persimilis*	CIS	CHILE	29/11/2017	1	*Flamingolepis* sp. 1
4	*Artemia persimilis*	CIS	CHILE	29/11/2017	1	*Flamingolepis* sp. 1
5	*Artemia persimilis*	CIS	CHILE	29/11/2017	1	*Flamingolepis* sp. 1
6	*Artemia persimilis*	CIS	CHILE	29/11/2017	1	*Flamingolepis* sp. 1
7	*Artemia persimilis*	CIS	CHILE	29/11/2017	1	*Flamingolepis* sp. 1
8	*Artemia persimilis*	CIS	CHILE	29/11/2017	1	*Flamingolepis* sp. 1
9	*Artemia persimilis*	CIS	CHILE	29/11/2017	1	*Flamingolepis* sp. 1
10	*Artemia persimilis*	CIS	CHILE	29/11/2017	1	*Flamingolepis* sp. 1
11	*Artemia persimilis*	CIS	CHILE	29/11/2017	1	*Flamingolepis* sp. 1
12	*Artemia persimilis*	CIS	CHILE	29/11/2017	1	*Flamingolepis* sp. 1
13	*Artemia persimilis*	CIS	CHILE	29/11/2017	1	*Flamingolepis* sp. 1
14	*Artemia persimilis*	CIS	CHILE	29/11/2017	1	*Flamingolepis* sp. 1
15	*Artemia persimilis*	CIS	CHILE	29/11/2017	1	*Flamingolepis* sp. 1
16	*Artemia persimilis*	CIS	CHILE	29/11/2017	1	*Flamingolepis* sp. 1
17	*Artemia persimilis*	CIS	CHILE	29/11/2017	1	*Flamingolepis* sp. 1
18	*Artemia persimilis*	CIS	CHILE	29/11/2017	1	*Flamingolepis* sp. 1
19	*Artemia persimilis*	CIS	CHILE	29/11/2017	1	*Flamingolepis* sp. 1
20	*Artemia persimilis*	CIS	CHILE	29/11/2017	1	*Flamingolepis* sp. 1
21	*Artemia persimilis*	CIS	CHILE	29/11/2017	1	*Flamingolepis* sp. 1
22	*Artemia persimilis*	CIS	CHILE	29/11/2017	1	*Flamingolepis* sp. 1
23	*Artemia persimilis*	CIS	CHILE	29/11/2017	1	*Flamingolepis* sp. 1
24	*Artemia persimilis*	CIS	CHILE	29/11/2017	1	*Flamingolepis* sp. 1
25	*Artemia persimilis*	CIS	CHILE	29/11/2017	1	*Flamingolepis* sp. 1
26	*Artemia persimilis*	CIS	CHILE	29/11/2017	1	*Flamingolepis* sp. 1
27	*Artemia persimilis*	CIS	CHILE	29/11/2017	1	*Flamingolepis* sp. 1

28	*Artemia persimilis*	CIS	CHILE	29/11/2017	1	*Flamingolepis* sp. 1
29	*Artemia persimilis*	CIS	CHILE	29/11/2017	1	*Flamingolepis* sp. 1
30	*Artemia persimilis*	CIS	CHILE	29/11/2017	1	*Confluaria podicipina*
31	*Artemia persimilis*	CIS	CHILE	29/11/2017	1	*Confluaria podicipina*
32	*Artemia persimilis*	CIS	CHILE	29/11/2017	1	*Confluaria podicipina*
33	*Artemia persimilis*	CIS	CHILE	29/11/2017	1	*Confluaria podicipina*
34	*Artemia persimilis*	CIS	CHILE	29/11/2017	1	*Fimbriarioides* sp.
35	*Artemia persimilis*	CIS	CHILE	29/11/2017	1	*Fimbriarioides* sp.
36	*Artemia persimilis*	CIS	CHILE	29/11/2017	1	*Fimbriarioides* sp.
37	*Artemia persimilis*	CIS	CHILE	29/11/2017	1	*Fimbriarioides* sp.
38	*Artemia persimilis*	CIS	CHILE	17/04/2018	1	*Fimbriarioides* sp.
39	*Artemia persimilis*	AMA	CHILE	28/11/2011	1	*Confluaria podicipina*
40	*Artemia persimilis*	AMA	CHILE	28/11/2011	1	*Confluaria podicipina*
41	*Artemia persimilis*	AMA	CHILE	28/11/2011	1	*Confluaria podicipina*
42	*Artemia persimilis*	AMA	CHILE	27/11/2017	1	*Confluaria podicipina*
43	*Artemia persimilis*	AMA	CHILE	27/11/2017	1	*Confluaria podicipina*
44	*Artemia persimilis*	AMA	CHILE	18/04/2018	1	*Fimbriarioides* sp.
45	*Artemia persimilis*	AMA	CHILE	18/04/2018	1	*Fimbriarioides* sp.
46	*Artemia franciscana*	BNE	CHILE	31/05/2018	1	*Flamingolepis* sp. 1
47	*Artemia franciscana*	BNE	CHILE	31/05/2018	1	*Flamingolepis* sp. 1
48	*Artemia franciscana*	BNE	CHILE	31/05/2018	1	*Flamingolepis* sp. 1
49	*Artemia franciscana*	BNE	CHILE	31/05/2018	1	*Flamingolepis* sp. 1
50	*Artemia franciscana*	BNE	CHILE	31/05/2018	1	*Flamingolepis* sp. 1
51	*Artemia franciscana*	BNE	CHILE	31/05/2018	1	*Flamingolepis* sp. 1
52	*Artemia franciscana*	BNE	CHILE	31/05/2018	1	*Flamingolepis* sp. 1
53	*Artemia franciscana*	BNE	CHILE	31/05/2018	1	*Flamingolepis* sp. 1
54	*Artemia franciscana*	BNE	CHILE	31/05/2018	1	*Flamingolepis* sp. 1
55	*Artemia franciscana*	BNE	CHILE	31/05/2018	1	*Flamingolepis* sp. 1
56	*Artemia franciscana*	TEB	CHILE	02/06/2018	1	*Flamingolepis* sp. 1
57	*Artemia franciscana*	TEB	CHILE	02/06/2018	1	*Flamingolepis* sp. 1
58	*Artemia franciscana*	TEB	CHILE	02/06/2018	1	*Flamingolepis* sp. 1
59	*Artemia franciscana*	TEB	CHILE	02/06/2018	1	*Flamingolepis* sp. 1
60	*Artemia franciscana*	TEB	CHILE	02/06/2018	1	*Flamingolepis* sp. 1
61	*Artemia franciscana*	TEB	CHILE	02/06/2018	1	*Flamingolepis* sp. 1
62	*Artemia franciscana*	TEB	CHILE	02/06/2018	1	*Flamingolepis* sp. 1

63	*Artemia franciscana*	TEB	CHILE	02/06/2018	1	*Flamingolepis* sp. 1
64	*Artemia franciscana*	TEB	CHILE	02/06/2018	1	*Flamingolepis* sp. 1
65	*Artemia franciscana*	TEB	CHILE	02/06/2018	1	*Flamingolepis* sp. 1
66	*Artemia franciscana*	TEB	CHILE	02/06/2018	1	*Flamingolepis* sp. 2
67	*Artemia parthenogenetica*	BRP	SPAIN	30/10/2007	1	*Flamingolepis liguloides*
68	*Artemia parthenogenetica*	BRP	SPAIN	30/10/2007	1	*Flamingolepis liguloides*
69	*Artemia parthenogenetica*	BRP	SPAIN	30/10/2007	2	*Flamingolepis liguloides*
70	*Artemia parthenogenetica*	BRP	SPAIN	30/10/2007	3	1 *Flamingolepis liguloides* + 2 *Flamingolepis flamingo*
71	*Artemia parthenogenetica*	BRP	SPAIN	30/10/2007	3	2 *Flamingolepis liguloides* + 1 *Confluaria podicipina*
72	*Artemia parthenogenetica*	BRP	SPAIN	30/10/2007	4	3 *Flamingolepis liguloides* + 1 *Wardium stellorae*
73	*Artemia parthenogenetica*	BRP	SPAIN	30/10/2007	5	*Flamingolepis liguloides*
74	*Artemia parthenogenetica*	BRP	SPAIN	30/10/2007	2	*Flamingolepis liguloides*
75	*Artemia parthenogenetica*	BRP	SPAIN	30/10/2007	2	*Flamingolepis liguloides*
76	*Artemia parthenogenetica*	BRP	SPAIN	30/10/2007	1	*Flamingolepis liguloides*
77	*Artemia parthenogenetica*	BRP	SPAIN	30/10/2007	1	*Flamingolepis flamingo*
78	*Artemia parthenogenetica*	BRP	SPAIN	30/10/2007	3	2 *Flamingolepis liguloides* + 1 *Flamingo flamingo*
79	*Artemia parthenogenetica*	BRP	SPAIN	30/10/2007	3	2 *Flamingolepis liguloides* + 1 *Flamingolepis flamingo*
80	*Artemia parthenogenetica*	SPP	SPAIN	30/10/2007	12	2 *Flamingolepis liguloides* + 10 *Confluaria podicipina*
81	*Artemia parthenogenetica*	SPP	SPAIN	30/10/2007	2	1 *Flamingolepis liguloides* + 1 *Confluaria podicipina*
82	*Artemia parthenogenetica*	SPP	SPAIN	30/10/2007	1	*Confluaria podicipina*
83	*Artemia parthenogenetica*	SPP	SPAIN	30/10/2007	2	*Confluaria podicipina*
84	*Artemia franciscana*	GSL	USA	20/09/2009	1	*Confluaria podicipina*
85	*Artemia franciscana*	GSL	USA	20/09/2009	1	*Confluaria podicipina*
86	*Artemia franciscana*	GSL	USA	20/09/2009	1	*Confluaria podicipina*
87	*Artemia franciscana*	GSL	USA	20/09/2009	1	*Confluaria podicipina*
88	*Artemia franciscana*	GSL	USA	20/09/2009	1	*Confluaria podicipina*
89	*Artemia franciscana*	GSL	USA	20/09/2009	1	*Confluaria podicipina*
90	*Artemia franciscana*	GSL	USA	20/09/2009	1	*Confluaria podicipina*
91	*Artemia franciscana*	GSL	USA	20/09/2009	1	*Confluaria podicipina*
92	*Artemia franciscana*	GSL	USA	20/09/2009	1	*Confluaria podicipina*
93	*Artemia franciscana*	GSL	USA	20/09/2009	1	*Confluaria podicipina*
94	*Artemia franciscana*	GSL	USA	20/09/2009	1	*Confluaria podicipina*
95	*Artemia franciscana*	GSL	USA	20/09/2009	2	*Confluaria podicipina*
96	*Artemia franciscana*	GSL	USA	20/09/2009	2	*Confluaria podicipina*
97	*Artemia franciscana*	GSL	USA	20/09/2009	1	*Confluaria podicipina*

98	*Artemia franciscana*	GSL	USA	20/09/2009	1	*Confluaria podicipina*
99	*Artemia franciscana*	GSL	USA	22/06/2016	1	*Confluaria podicipina*
100	*Artemia franciscana*	GSL	USA	22/06/2016	1	*Confluaria podicipina*
101	*Artemia franciscana*	GSL	USA	22/06/2016	1	*Hymenolepis californicus*
102	*Artemia franciscana*	GSL	USA	22/06/2016	2	*Hymenolepis californicus*
103	*Artemia franciscana*	GSL	USA	22/06/2016	2	*Confluaria podicipina* + *Hymenolepis californicus*
104	*Artemia franciscana*	GSL	USA	22/06/2016	1	*Hymenolepis californicus*
105	*Artemia franciscana*	GSL	USA	22/06/2016	1	*Confluaria podicipina*
106	*Artemia franciscana*	GSL	USA	22/06/2016	3	*Confluaria podicipina*
107	*Artemia franciscana*	GSL	USA	22/06/2016	2	*Confluaria podicipina*
108	*Artemia franciscana*	GSL	USA	22/06/2016	1	*Hymenolepis californicus*
109	*Artemia franciscana*	GSL	USA	22/06/2016	1	*Hymenolepis californicus*
110	*Artemia franciscana*	GSL	USA	22/06/2016	2	*Confluaria podicipina* + *Hymenolepis californicus*
111	*Artemia franciscana*	GSL	USA	22/06/2016	2	*Confluaria podicipina* + *Hymenolepis californicus*
112	*Artemia franciscana*	GSL	USA	22/06/2016	2	*Confluaria podicipina*
113	*Artemia franciscana*	GSL	USA	21/07/2017	3	*2 Confluaria podicipina* + *Hymenolepis californicus*
114	*Artemia franciscana*	GSL	USA	21/07/2017	3	2 *Confluaria podicipina* + 1 *Hymenolepis californicus*
115	*Artemia franciscana*	GSL	USA	21/07/2017	1	*Hymenolepis californicus*
116	*Artemia franciscana*	GSL	USA	21/07/2017	1	*Hymenolepis californicus*
117	*Artemia franciscana*	GSL	USA	21/07/2017	1	*Hymenolepis californicus*
118	*Artemia franciscana*	GSL	USA	21/07/2017	1	*Hymenolepis californicus*
119	*Artemia franciscana*	GSL	USA	21/07/2017	1	*Hymenolepis californicus*
120	*Artemia franciscana*	GSL	USA	21/07/2017	2	*Confluaria podicipina*
