# Supplementary material for: Phylogenetic Relationships of Avian Cestodes from Brine Shrimp and Congruence with Larval Morphology
Source: Animals (Basel). 2024 Jan 25;14(3):397. doi: 10.3390/ani14030397 (PMC10854740; doi:10.3390/ani14030397)
Supplement: Supplementary file 1 [file animals-14-00397-s001.zip › Table S2_Sequences for primer design.pdf]

Table S2. Details of 18S rDNA sequences of hymenolepidid cestodes used for primer design. Final hosts are also indicated. WB, waterbirds; B, other birds; M, mammals

Gen Bank accesion number	Cestode	Final Host	Country	18S ribosomal RNA gene	Sequence size (bp)	Reference
KJ710320	*Coronacanthus integrus*	*Neomys fodiens* (M)	Bulgaria	partial	2,005	[29]
KJ710321	*Coronacanthus magnihamatus*	*Neomys fodiens* (M)	Bulgaria	partial	1,996	[29]
KJ710319	*Coronacanthus omissus*	*Neomys fodiens* (M)	Bulgaria	partial	2,005	[29]
AF286982	*Fimbriaria* sp.	*Anas platyrhynchus* (WB)	USA	complete	2,137	[57]
JX173961	Hymenolepididae gen. sp.	*Haematopus unicolor* (WB)	New Zealand	partial	1,971	[59]
AF124475	*Hymenolepis diminuta*	*Rattus norvegicus* (M)		complete	2,174	[64]
AJ287525	*Hymenolepis microstoma*	*Mus* sp. (M)		complete	2,140	[65]
KY403995	*Hymenolepis microps*	*Lagopus lagopus* (B)		partial	2,233	[50]
AY193875	*Hymenolepis nana* (syn. *Rodentolepis nana*)	*Homo sapiens* (M)	USA	complete	2,244	[66]
AJ287587	*Wardoides nyrocae*	*Cygnus olor* (WB)	Scotland	complete	2,186	[65]

[29] Neov, B.; Vasileva, G.P.; Radoslavov, G.; Hristov, P.; Littlewood, D.T.J.; Georgiev, B.B. Phylogeny of hymenolepidids (Cestoda: Cyclophyllidea) from mammals: sequences of 18S rRNA and COI genes confirm major clades revealed by the 28S rRNA analyses. *J. Helminthol.* **2021**, *95*. e23. DOI: 10.1017/S0022149X21000110

[57] Olson, P.D.; Littlewood, D.T.J.; Bray, R.A.; Mariaux, J. Interrelationships and evolution of the tapeworms (Platyhelminthes: Cestoda). *Mol. Phylogenet. Evol.* **2001**, *19*, 443-467. DOI: 10.1006/mpev.2001.0930

[59] Presswell, B.; Melville, D.S.; Randhawa, H.S. Tapeworm bolus expelled from New Zealand variable oystercatchers (*Haematopus unicolor*) during handling: first record of this phenomenon in wild birds, and a global checklist of *Haematopus* cestode parasites. *Parasitol. Res.* **2012**, *111*, 2455-2460. DOI: 10.1007/s00436-012-2992-9

[64] Olson, P.D.; Caira, J.N. Evolution of the major lineages of tapeworms (Platyhelminthes: Cestoidea) inferred from 18S ribosomal DNA and elongation factor-1α. *J. Parasitol.* **1999**, *85*, 1134-1159. DOI: 10.2307/3285679

[65] Littlewood, D.T.J.; Olson, P.D. Small subunit rDNA and the Platyhelminthes: signal, noise, conflict, and compromise. In *Interrelationships of the Platyhelminthes*; Littlewood, D.T.J., Bray, R.A., Eds.; Taylor and Francis: London, United Kingdom, **2001**; pp. 262-278.

[50] Pistone, D.; Lindgren, M.; Holmstad, P.; Ellingsen, N.K.; Kongshaug, H.; Nilsen, F.; Skorping, A. The role of chewing lice (Phthiraptera: Philopteridae) as intermediate hosts in the transmission of *Hymenolepis microps* (Cestoda: Cyclophyllidea) from the willow ptarmigan *Lagopus lagopus* (Aves: Tetraonidae). *J. Helminth.* **2018**, *92*, 49-55. DOI: 10.1017/S0022149X17000141

[66] Olson, P.D.; Yoder, K.; Fajardo LG, L.F.; Marty, A.M.; van de Pas, S.; Olivier, C.; Relman, D.A. Lethal invasive cestodiasis in immunosuppressed patients. *J. Infect, Dis.* **2003**, *187*, 1962-1966.
