# Supplementary material for: Phylogenetic Relationships of Avian Cestodes from Brine Shrimp and Congruence with Larval Morphology
Source: Animals (Basel). 2024 Jan 25;14(3):397. doi: 10.3390/ani14030397 (PMC10854740; doi:10.3390/ani14030397)
Supplement: Supplementary file 1 [file animals-14-00397-s001.zip › Table S3_Matrix genetic divergence.pdf]

Table S3. Estimates of genetic divergence over sequence pairs between taxa. The number of base differences per site from averaging over all sequence pairs between taxa are shown on the left side from diagonal. The right side from the diagonal shows the rate of variation among sites using the Tamura-Nei model with a gamma distribution. Both analyses involved 28 nucleotide sequences. All positions containing gaps were removed, i.e., complete deletion. There were a total of 988 positions in the final dataset.

	1	2	3	4	5	6	7	8	9	10	11	12	13	14	15	16	17	18	19
1 *Flamingolepis* sp. 1		0.031	0.036	0.127	0.114	0.117	0.103	0.103	0.128	0.125	0.126	0.109	0.104	0.105	0.125	0.100	0.117	0.109	0.154
2 *Flamingolepis* sp. 2	0.029		0.007	0.125	0.103	0.107	0.100	0.098	0.120	0.126	0.122	0.095	0.098	0.104	0.126	0.102	0.111	0.106	0.151
3 *Flamingolepis liguloides*	0.034	0.007		0.134	0.108	0.116	0.101	0.099	0.121	0.135	0.131	0.101	0.103	0.112	0.135	0.110	0.116	0.111	0.154
4 *Fimbriarioides* sp.	0.107	0.105	0.111		0.082	0.079	0.100	0.100	0.100	0.010	0.013	0.074	0.065	0.101	0.010	0.075	0.092	0.079	0.146
5 *Confluaria podicipina*	0.097	0.089	0.093	0.073		0.039	0.076	0.076	0.096	0.085	0.077	0.048	0.046	0.073	0.085	0.046	0.057	0.048	0.138
6 *Hymenolepis californicus*	0.099	0.092	0.098	0.070	0.037		0.073	0.073	0.084	0.079	0.075	0.029	0.039	0.063	0.079	0.042	0.048	0.049	0.134
7 Hymenolepididae sp. 2	0.089	0.087	0.088	0.087	0.068	0.066		0.002	0.102	0.103	0.095	0.071	0.069	0.076	0.103	0.064	0.068	0.068	0.145
8 Hymenolepididae sp. 3	0.089	0.085	0.086	0.087	0.068	0.066	0.002		0.099	0.103	0.095	0.071	0.068	0.073	0.103	0.064	0.068	0.068	0.145
9 Hymenolepididae sp. 1	0.107	0.101	0.102	0.087	0.083	0.074	0.088	0.086		0.100	0.098	0.079	0.081	0.106	0.100	0.084	0.100	0.095	0.146
10 *Fimbriaria teresae*	0.106	0.106	0.112	0.010	0.075	0.070	0.089	0.089	0.087		0.008	0.076	0.066	0.106	0.000	0.074	0.093	0.081	0.143
11 *Fimbriaria fasciolaris*	0.106	0.103	0.109	0.013	0.069	0.067	0.083	0.083	0.085	0.008		0.074	0.064	0.104	0.008	0.069	0.084	0.074	0.137
12 Hymenolepididae sp. 4	0.093	0.083	0.087	0.066	0.045	0.027	0.064	0.064	0.070	0.068	0.066		0.045	0.075	0.076	0.045	0.060	0.049	0.123
13 *Hymenolepis microps*	0.090	0.085	0.089	0.059	0.043	0.036	0.062	0.062	0.072	0.060	0.058	0.041		0.069	0.066	0.033	0.038	0.039	0.128
14 *Wardoides nyrocae*	0.091	0.090	0.096	0.087	0.065	0.057	0.068	0.066	0.090	0.091	0.089	0.067	0.062		0.106	0.073	0.077	0.078	0.143
15 *Fimbriaria* sp.	0.106	0.106	0.112	0.010	0.075	0.070	0.089	0.089	0.087	0.000	0.008	0.068	0.060	0.091		0.074	0.093	0.081	0.143
16 *Hymenolepis diminuta*	0.087	0.088	0.094	0.068	0.043	0.039	0.059	0.059	0.075	0.067	0.063	0.042	0.031	0.066	0.067		0.038	0.027	0.125
17 *Hymenolepis microstoma*	0.099	0.095	0.099	0.080	0.052	0.044	0.062	0.062	0.086	0.081	0.074	0.055	0.035	0.069	0.081	0.035		0.036	0.126
18 *Coronacanthus magnihamatus*	0.093	0.091	0.095	0.071	0.045	0.045	0.062	0.062	0.083	0.072	0.067	0.045	0.036	0.069	0.072	0.026	0.034		0.125
19 *Dilepis undula*	0.126	0.124	0.127	0.120	0.116	0.112	0.120	0.120	0.120	0.118	0.114	0.104	0.108	0.118	0.118	0.106	0.107	0.106	
